# *Reph*, a Regulator of Eph Receptor Expression in the *Drosophila melanogaster* Optic Lobe

**DOI:** 10.1371/journal.pone.0037303

**Published:** 2012-05-16

**Authors:** Richard E. Dearborn, Yong Dai, Brian Reed, Tamar Karian, Jessica Gray, Sam Kunes

**Affiliations:** 1 Department of Molecular and Cellular Biology, Harvard University, Cambridge, Massachusetts, United States of America; 2 Department of Pharmaceutical Sciences, Albany College of Pharmacy and Health Sciences, Albany, New York, United States of America; National Institutes of Health (NIH), United States of America

## Abstract

Receptors of the Eph family of tyrosine kinases and their Ephrin ligands are involved in developmental processes as diverse as angiogenesis, axon guidance and cell migration. However, our understanding of the Eph signaling pathway is incomplete, and could benefit from an analysis by genetic methods. To this end, we performed a genetic modifier screen for mutations that affect Eph signaling in *Drosophila melanogaster*. Several dozen loci were identified on the basis of their suppression or enhancement of an eye defect induced by the ectopic expression of Ephrin during development; many of these mutant loci were found to disrupt visual system development. One modifier locus, *reph* (regulator of eph expression), was characterized in molecular detail and found to encode a putative nuclear protein that interacts genetically with Eph signaling pathway mutations. Reph is an autonomous regulator of Eph receptor expression, required for the graded expression of Eph protein and the establishment of an optic lobe axonal topographic map. These results reveal a novel component of the regulatory pathway controlling expression of *eph* and identify *reph* as a novel factor in the developing visual system.

## Introduction

Bi-directional signaling through receptor tyrosine kinases of the Eph family and their Ephrin ligands contributes to diverse processes during and after development including the establishment of topographic axon projections in the visual system [1], [2], cell migration [3], [4], vascular development [5] and long-term potentiation [6]–[8]. Crucial to deciphering the mechanisms by which this signaling pathway mediates such diverse and complex processes is a complete catalog of its pathway components. Substantial progress towards this end has been made by biochemical approaches [9]. Activation of Eph receptors and transmembrane B-class Ephrin ligands regulates cytoskeletal dynamics through the recruitment one of several SH2/SH3 adaptor proteins [10]–[12] and PDZ-domain proteins [13]. Ras superfamily GTPase activity [14]–[16] and crosstalk with other signaling pathways including MAPK [17], [18], PI-3 kinase [19] and heterotrimeric G proteins [13] are involved. Nonetheless, many effector molecules may have been missed by these approaches. A complementary approach, utilizing genetic analysis, offers the possibility of additional insights into Eph signal transduction. We have undertaken this approach in *D. melanogaster*.

The diverse activities and functional redundancy of the large vertebrate Eph and Ephrin families complicates pathway studies. The *D. melanogaster* genome, in contrast, encodes only a single Eph receptor and Ephrin ligand [20]. Previous work has demonstrated a conserved role for the *D. melanogaster* Eph pathway in the establishment of adult visual system axonal topography [21] and axon guidance within the mushroom body [22]. Within the visual system, Eph is expressed in a gradient, reminiscent of its graded retinal expression required for vertebrate retinotectal map formation, and controls the dorsoventral patterning of cortical axons that project centripetally to form the medulla neuropil [21]. Within the olfactory system, Eph is expressed by mushroom body neurons throughout development and Eph signaling regulates the guidance of individual axon branches within the mushroom body [22]. In light of these observations and the diversity of its resources for genetic analysis, *D. melanogaster* should serve as a valuable model system in which to identify novel components of the Eph pathway.

The *D. melanogaster* eye has often been used as an assay in genetic screens, including those for components of receptor tyrosine kinase (RTK) pathways [23]–[25]. Each ommatidium of the approximately 750 that comprise the compound eye is composed of the same complement of cells: eight photoreceptor neurons (R-cells) and a set of non-neuronal accessory cells, which includes lens-secreting cone cells and pigment cells [26]–[28]. The photoreceptor neurons project retinotopically into distinct regions of the optic lobes: R1–R6 axons terminate in the lamina while R7 and R8 axons terminate in different layers of the medulla [28]. The finely ordered structure of the *D. melanogaster* visual system gives rise to a neurocrystalline lattice, which is perturbed by changes in cell numbers, altered cell differentiation or aberrant axon projections. These types of developmental defects give rise to visible phenotypes that are well-suited to enhancer/suppressor screens. We utilized this approach, conducting a genetic screen for modifiers of an eye defect associated with the ectopic expression of Ephrin during eye development. More than two-dozen essential loci that either enhanced or suppressed this defect were identified. We also describe the isolation and molecular characterization of one such gene, *reph*, which is found to be a novel regulator of Eph expression in the developing *D. melanogaster* nervous system.

## Materials and Methods

### Modifier Screen for Eph Signaling Mutants

The eye phenotype that served as the basis for the modifier screen was produced by *sevenless2-GAL4*, *UAS-ephrin*. Stable stocks of *sevenless2-GAL4*, *UAS-ephrin* were generated by recombination and balanced over *CyO*, hereafter referred to as SE (*sevenless2-GAL4*, *UAS-ephrin*), in preparation for the modifier screen. To match as closely as possible the genetic background of SE for the screen, *sevenless2-GAL4* animals were isogenized and tested for endogenous modifiers of the SE phenotype; no endogenous modifiers were present in the isogenized *sevenless2-GAL4* stock.

Isogenized *sevenless2-GAL4* males were mutagenized following a 12-hour starvation by transfer to vials containing sucrose-soaked cotton impregnated with 3 µg/ml of ethyl-nitrosourea (ENU). After 12 hours of feeding on the sucrose/ENU food source, mutagenized males were transferred, in two sequential rounds, to normal food for 4-hour periods to allow for grooming and removal of external ENU contamination and subsequently mated with SE females in bottles at a ratio of 5 mutagenized males per 50 SE females. A total of 50 mutagenized males were crossed to 500 SE females. After incubation at 25°C, F_1_ progeny were examined for the presence of dominant enhancers or suppressors of the SE phenotype and scored. Candidate lines were back-crossed to SE to track the mutation and expand the line. Mutations were localized to individual chromosomes by subsequent crosses to balancer stocks (*FM-7* for the X-chromosome, *y^+^CyO* for chromosome II, *Tb/Sb* for chromosome III and *Ci^D^* for chromosome IV) and tracing of segregation of the modifier phenotype with the balancers. After localization to a particular chromosome, candidate mutants were back-crossed to SE to confirm the presence of the modifier mutation in the balanced stock.

### Mapping of Point Mutations to Gene Loci

To identify loci harboring point mutations in the Eph collection, lethal lines were crossed to chromosomal deficiency lines (Bloomington *Drosophila* Stock Center) and assessed for non-complementation. Successively smaller deficiencies were screened until the smallest obtainable deficiency-defined non-complementing region was identified. Next, P-element lethals within the target region were crossed to the SE modifier lethal and assessed for non-complementation. For SE modifier lethals in which available P-element lines failed to resolve the locus, local P-elements were hopped (see below) to generate a collection of new regional-specific P-element insertions. These *de novo* P-element hops were then individually crossed to the SE modifier lethal under scrutiny and assessed for non-complementation. Once a non-complementing line was identified, the locus of insertion was determined by inverse PCR from the P-element ends (see below).

### P-element Insertional Mutagenesis and Inverse PCR Analysis of Insertion Loci

Quiescent P-elements were mobilized by crossing target lines (see below) to flies of the genotype [*w^1118^*; *sp*/*CyO*; Δ2-3,*Sb*/*TM6B*] and incubating at 25°C. In most cases, mobilization of resident P-elements by the Δ2-3 transposase involves duplication of the existing P-element followed by hopping of the duplicated element. Successfully induced hops therefore manifest themselves in F_1_ offspring as darker eye pigmentation owing to the presence of two copies of the *w^+^* marker carried by the P-element. For identification of the *reph* locus two lethal P-element lines, p[K08617]/*CyO* and p[K16918]/*CyO*, localized to a small deficiency non-complementing to the *reph^1^* mutant, were used as starting lines to produce a new collection of local hops. Individual F_1_ hops from these lines were crossed directly to balanced *reph^1^* mutants and assessed for non-complementation. Non-complementing lines were then subjected to inverse PCR analysis to determine the locus of insertion. For generation of the *ephrin* hypomorph, *ephrin^RS5^*, an *RS5* P-element insertion on chromosome IV [29] was mobilized. Candidate local hops mapping to chromosome IV were further analyzed by Southern blot of the *ephrin* locus. Insertions within the *ephrin* locus revealed by Southern blot were then subjected to inverse PCR to determine the precise location of the P-element insertion.

Inverse PCR was performed essentially as described (E.J. Rehm, BDGP, http://www.fruitfly.org/about/methods/inverse.pcr.html), but is summarized here. Flies were homogenized in 1× PBS/0.5 µg/ml Proteinase K (25 µl per fly) in eppendorf tubes, incubated at room temperature for 30 minutes and subsequently heat-inactivated at 100°C for 5 minutes. The homogenate was then spun down and the supernatant collected. 10 µl of genomic extract was then cut with one of three restriction enzymes: *Sau3A I*, *HinP1 I* or *Msp I*. Qiagen column-cleaned digests were then circularized and used as template for PCR under the following conditions: 40× cycles, 3 min extension, 50°C annealing temperature. The following primer pairs were used in the inverse PCR reactions: A) pLac1 5′ CACCCAAGGCTCTGCTCCCACATT 3′ & pLac4 5′ ACTGTGCGTTAGGTCCTGTTCATTGTT 3′ and B) Sp1 5′ ACACAACCTTTCCTCTCAACAA 3′ & Splac2 5′ GAATTCACTGGCCGTCGTTTTACAA 3′. Successfully amplified bands were gel purified using a Qiagen column, sub-cloned into Topo according to manufacturers' protocol (Invitrogen) and used to transform competent cells. Mini-preps (Qiagen) obtained from cells transformed with Topo vector containing inverse PCR inserts were used as template for sequencing reactions (ABI Prism BigDye Terminator Cycle Sequencing Ready Reaction Kit). Sequences obtained from inverse PCR fragments were then BLASTed against the *D. melanogaster* genome to identify the site of P-element insertion.

### Targeted Homologous Recombination to Generate Kinase-Dead Eph Animals: DNA constructs and fly crosses

Targeted homologous recombination was performed essentially as described by Rong and Golic [30]. A 6.0 kb *EcoRI* fragment of the *eph* genomic region was first cloned into pBS^(KS)^. Subsequently, an *I-SceI* recognition site was synthesized as two oligos and cloned into a unique site in exon 6 of the *eph* gene. The *I-Sce-I* modified *eph* genomic fragment was then cloned into the *Not I* site of the P-element vector pP[>*w^hs^* ^.^N>} to produce the donor construct pP[>*w^hs^* ^.^ *eph^KD^*>] for *eph* targeting. The donor construct was then transformed into flies [31]. Recombination was induced by crossing *y^1^w^1^*; [donor]/*y^+^CyO*; [70*FLP*], [70 *I-Sce1*]/*TM3*,*Sb*,*e* flies to *y^1^w^67c23^* flies and heat shocking. Progeny were then screened for *w^+^CyO* or changed *w^+^* eye color and test-crossed to map the *w^+^* marker. Animals with *w^+^* mapping to the chromosome IV were examined via Southern blot to verify the targeting event.

### Targeted Homologous Recombination to Generate Kinase-Dead Eph Animals: Molecular Analyses of Targeting Efficacy

Southern blot analyses were performed according to standard protocols [32] using probes targeting the *I-Sce-I* cut site of the donor construct. In addition, RT-PCR experiments were performed on mRNA isolated from putative *eph^KD^* animals to verify the presence of predicted truncated transcripts.

### Generation and misexpression of *P[UAS-reph]* and P[UAS-ephrin] transgenes

A 2 kb *EcoRI/XhoI* fragment of *reph* containing the entire coding region for the 401 amino acid isoform was excised from a pOT2 cloning vector (GH05923, Drosophila Genomics Resource Center, Indiana University) and inserted into the transformation vector pUAST [33] to generate *P[UAS-reph]*. Likewise, for *P[UAS-ephrin]*, the full-length coding region was amplified by PCR and inserted into pUAST. Transformation of *D. melanogaster* was performed as described by Rubin and Spradling [31]. Patterned expression of the *P[UAS-reph]* and *P[UAS-ephrin]* transgenes was accomplished using the *UAS-GAL4* system [33]. All crosses were grown at 25°C and immunohistochemical analyses performed on late third instar larvae. The following crosses were used: (A) *y*,*hsFLP_122_*;;*UAS-reph* X *y*,*w*; *UAS-CD8*::*GFP*, *tub*_α1_.*y* ^+^, *CD2*>*GAL4*/*y* ^+^ *CyO*, (B) *sevenless2-GAL4 X UAS-ephrin*, (C) *sevenless2-GAL4*, *UAS-ephrin/y* ^+^ *CyO* X *UAS-reph* (‘>’ indicates the position of an FRT site in the respective construct).

### Mosaic Analysis of *reph* function in the Developing Optic Lobe

Mosaic analysis was carried out as described by Xu and Rubin [34]. Larvae were subjected to heat shock at 37°C for 60–75 minutes 24–36 hours after hatching to induce expression of an hsFLP transgene. After growth at 20°C, larvae were dissected at late larval third instar stage and processed for immunohistochemical analysis. The following crosses and strains were used in the experiments described: *y*,*hsFLP_122_*; P{*arm-lacZ*}42D, P{FRT}42D/P{*y* ^+^},*CyO* X *y*,*w*; P{*lacW*}*reph*, P{FRT}43D/P{*y* ^+^}, *CyO*

### Immunocytochemistry

Immunocytochemically staining was performed essentially as described in [35]. Primary antibodies were used at the following dilutions: rabbit anti-Eph 1∶200 [21], rabbit anti-Ephrin 1∶200 [36], goat FITC or Cy3 anti-HRP (Cappel) 1∶200, mouse anti-βgal (Promega) 1∶100. Secondary antibodies were used at the following dilutions: Cy3 or Cy5 goat anti-mouse (Jackson Immunochemical, Inc.) 1∶100, Cy3 or Cy5-goat anti-rabbit (Jackson) 1∶500, HRP-conjugated goat anti-mouse IgG (Jackson) 1∶100. Specimens were viewed on a Zeiss LSM510 META confocal microscope.

### RNA localization by tissue *in situ* hybridization

*In situ* hybridization was performed on larval third instar specimens as referenced in [21]. A pOT2 vector containing a full-length *reph* cDNA (GH05923, Drosophila Genomics Resource Center, Indiana University) was used as template to prepare both sense (*BglII* linearized, Sp6 RNA polymerase transcription) and antisense (*XhoI* linearized, T7 RNA polymerase transcription) digoxigenin-labeled probes (Riboprobe Combination System-Sp6/T7, Promega). Hybridized specimens were developed following incubation with anti-digoxigenin-alkaline phosphatase conjugated antibodies (mouse anti-digoxin, Sigma). Bright-field images were captured with a CCD camera.

## Results

### Eph and Ephrin Expression Patterns in the Developing Visual System

**Figure 1:**
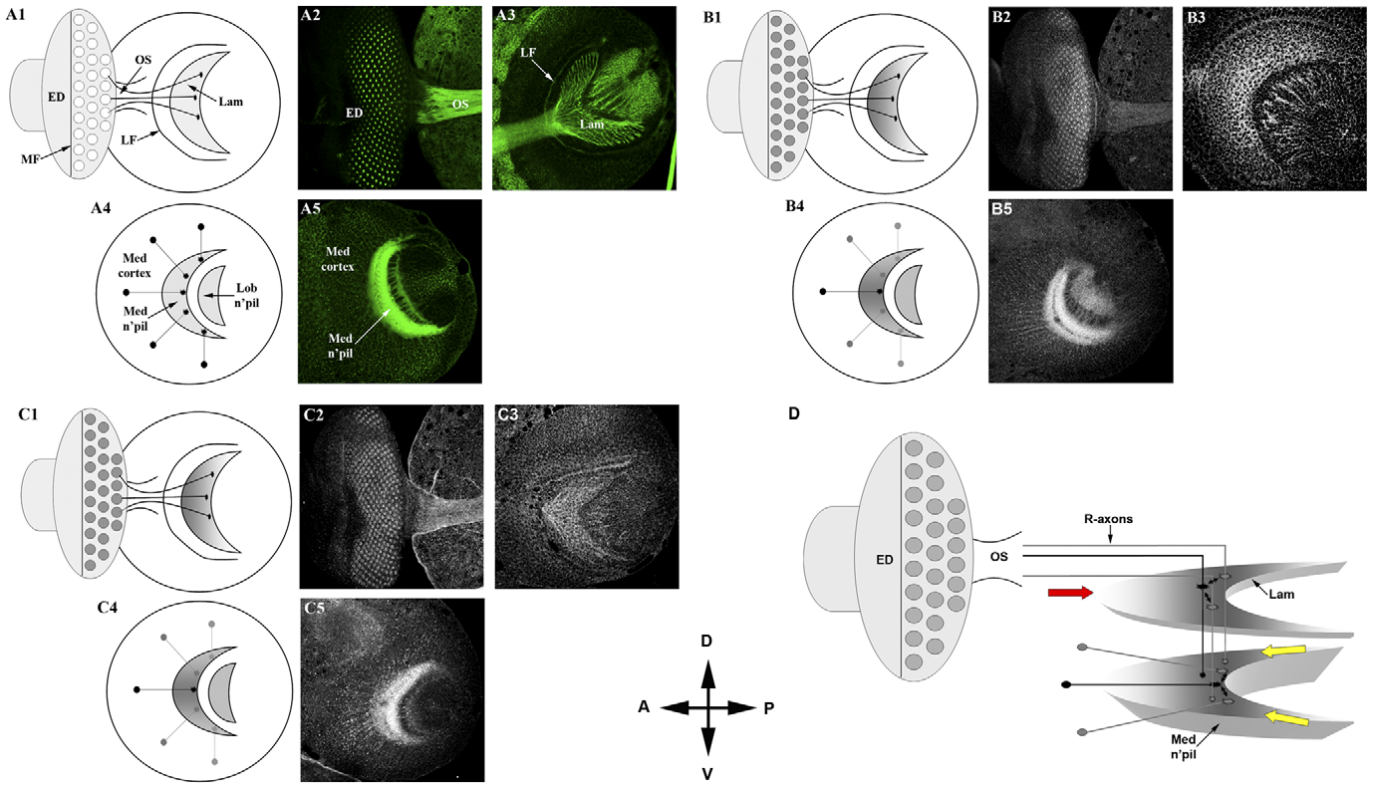
Eph and ephrin expression patterns in the D. melanogaster larval third instar optic lobe. A1–A5) anti-HRP staining (green) reveals overall optic lobe architecture. Posterior to the morphogenetic furrow (MF), retinal axons of ommatidia (small circles, A1) in the developing eye disk (ED; A2) project through the optic stalk (OS; A2) to topographically innervate two target regions in the brain: the lamina (Lam; demarcated by the lamina furrow, LF; A3) and the medulla (A5), which lies proximal to the lamina. Axons of medulla cortical neurons (Med cortex; A5) also project topographically to form a crescent-shaped neuropil (Med n'pil; A5). Schematic representations of optic lobe architecture are shown in A1 (eye and lamina) and A4 (medulla). B1-B5) anti-Eph staining (grayscale). Across the ED, Eph expression appears uniform posterior to the MF. Within the Lam, Eph expression is highest at the posterior midline and lowest at the dorsal-ventral margins of the anterior (B2, B3; depicted schematically in B1). Within the medulla, Eph expression is again highest at the midline, diminishing along the dorsal-ventral axis (B5; depicted schematically in B4). C1–C5) anti-Ephrin staining (grayscale) reveals uniform expression of Ephrin across the developing ED (C2). Within the Lam Ephrin expression appeared relatively uniform (C3). Ephrin-specific expression patterns in the developing ED and Lam are depicted schematically in C1. Within the medulla, Ephrin expression mirrors that of Eph: highest at the midline, diminishing along the dorsal-ventral axis (C5; depicted schematically in C4). D) Summary of Eph/Ephrin expression patterns in the developing visual system (horizontal perspective, which depicts the spatial relationship of the lamina and medulla target fields). Anterior (red arrow) and dorsoventral margins (yellow arrows) are indicated. All image panels were of late third larval instar stage brains stained for HRP (anti-HRP, A2, A3, A5), Eph (anti-Eph, B2, B3, B5) or Ephrin (anti-Ephrin, C2, C3, C5). Note that A2, B2, and C2 are the same image for which individual channels have been displayed. All other images represent distinct specimens. Dorsal (D), ventral (V), anterior (A) and posterior (P) orientations (central compass) are identical for all panels.

We have previously shown that the Eph protein is expressed on the axons of photoreceptor neurons, medulla and lobula cortical neurons and the membranes of lamina neuron precursors. The medulla cortex and axonal projections establish a three-dimensional neuropil that provides topographically arrayed synaptic targets for R7 and R8 photoreceptor axons and lamina axons. Eph antigen on medulla axons displayed a dorsoventral gradient, high at the midline toward the dorsal and ventral poles (Figure 1; [21]). Moreover, we found that disturbing this gradient resulted in topographic medulla axon targeting defects [21]. In contrast, Eph antigen on photoreceptor axons appeared uniform across the dorsoventral axis of their retinotopic projections into the brain.

The *D. melanogaster* genome also encodes a single Ephrin ortholog, which is most similar to vertebrate Ephrin B class ligands [36]. While RNAi interference approaches have suggested a role for Eph/Ephrin signaling in neuronal development, notably within the embryonic CNS [21], [36], analyses of Eph null mutants did not detect embryonic CNS defects but rather a specific role for Eph/Ephrin signaling in the developing mushroom body [22]. The disparity between these results suggests a complex role for Eph/Ephrin signaling during *Drosophila* development that would benefit from additional characterization, specifically analyses of Ephrin expression patterns within the larval brain.

Like Eph, the expression of its ligand Ephrin was uniform across the dorsoventral columns of ommatidia posterior to the morphogenetic furrow (Figures 1-B2 and 1-C2). Within the lamina, retinal axons distribute themselves retinotopically along the dorsoventral and anteroposterior axes, forming a crescent-shaped target field (Figure 1-A1 and 1-A3). Retinal axons arrive in this field in a posterior to anterior temporal order, in concert with the temporal dynamics of photoreceptor cell differentiation in the retina. The axons from each dorsoventral column of photoreceptor neurons arrives in the target field contemporaneously, and distribute themselves retinotopically on the dorsoventral axis of the lamina. Eph expressed on the membranes of developing lamina neurons displayed gradients on both axes; high dorsoventral midline and high posterior to low anterior (Figure 1-B3; [21]). Ephrin expression, in contrast, appeared relatively uniform (Figure 1-C3). In the medulla ganglion, the target for R7/R8 photoreceptor axons, cortical axons project topographically into a crescent-shaped neuropil, which requires cues conveyed by the pattern of Eph expression [21]. As was the case for the developing eye disc, Eph and Ephrin were expressed in similar gradients on axons of medulla cortical neurons—high midline, low dorso-ventral (Figures 1-B5 and 1-C5, respectively). Co-expression of Eph receptors and Ephrin ligands has been observed in a number of tissues [22], [37], [38] and suggests that the mechanism by which Eph/Ephrin signaling regulates axon guidance is more complex than simple gradient-mediated interactions. Such co-expression would permit both forward and reverse signaling within the same cell, as well as for both trans and cis regulation of Eph/Ephrin complexes [39]. Thus, the effects of Eph/Ephrin signaling within a given cell may depend on the coordination of these various regulatory possibilities. Given the extensive expression of Eph and Ephrin in the larval third instar visual system (summarized in Figure 1D) and observations that disruption of Eph expression affects axon topography [21] we reasoned that the developing *D. melanogaster* visual system might provide an amenable model to screen for components of the Eph/Ephrin signaling pathway.

### A Dominant Modifier Screen for Eph Pathway Components

**Figure 2:**
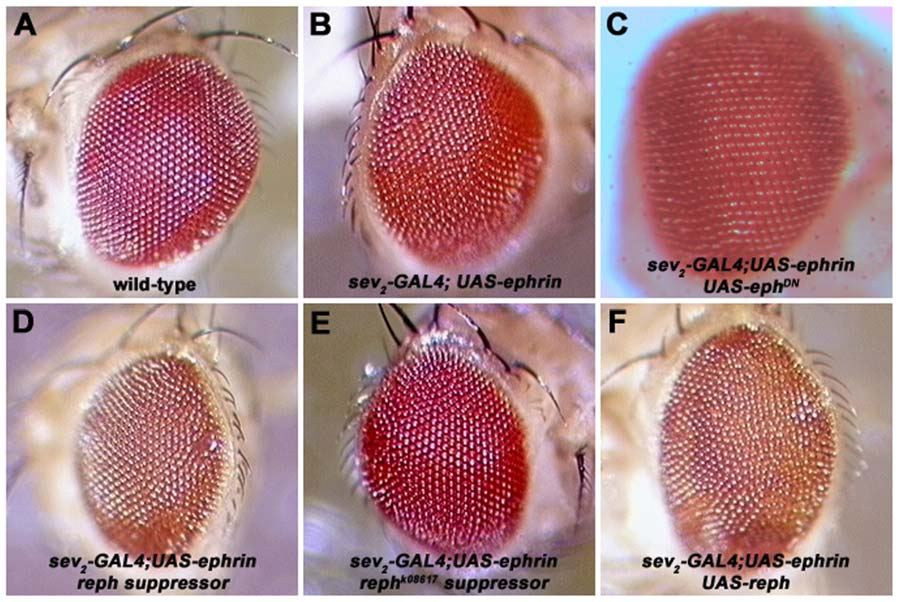
A genetic screen for Eph pathway signaling molecules. A) The wild-type adult eye displays a regular ommatidial lattice. B) The rough-eye phenotype generated by expression of *UAS-ephrin* under control of the *sevenless2-GAL4* driver (SE) used to screen for modifier mutations. C) Near-complete suppression of the SE phenotype by co-expression of a dominant-negative *eph* transgene (*eph^DN^*). D) Suppression of the SE phenotype by the *reph^1^* allele. E) Suppression of the SE phenotype by *reph^k8617^*. F) Co-expression of a *UAS-reph^+^* transgene enhances the SE phenotype. All images (20×) were acquired through a digital camera attached to a Zeiss Stemi SV11 stereo dissecting microscope. Images were captured using FPG3 software.

**Figure 3:**
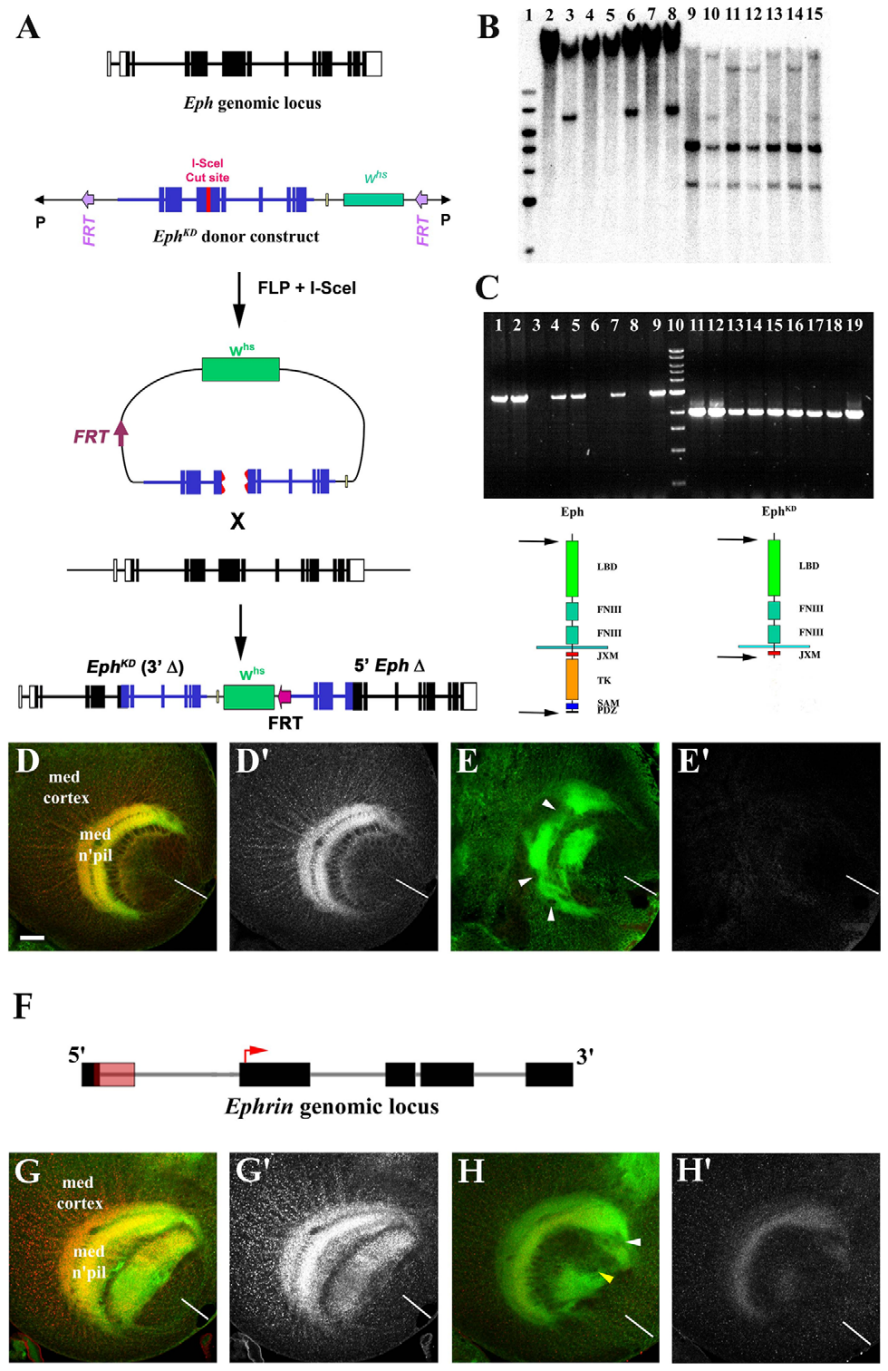
Isolation of *eph* and *ephrin* mutants. A) The 10.2 kb *eph* locus (topmost diagram) consists of 14 exons (boxes; white = UTRs, black = coding sequences) and 13 introns (lines), localized to the 102D2-D5 region of chromosome IV. The core donor construct (see Methods for details) consisted of *eph* genomic sequences (blue) lacking exons 1–4 (deleting the 5′UTR, start codon, signal sequence and a portion of the exoplasmic domain) and a 3′ region lacking the kinase domain and terminal 3′ sequences. An *I-SceI* site was engineered into the middle of exon 6 (red shading). The core construct was placed upstream of a white gene marker (*w^hs^*, green shading), the whole being bracketed by FLP recognition target sequences (FRT, purple shading) and inserted into the transformation vector. ‘Ends-in’ recombination induced by FLP and *I-SceI* resulted in partial tandem duplication of the *eph* locus (bottom-most diagram). B) Southern blot analysis of five candidate *eph^KD^* targeting events. Lane 1: molecular weight markers. Lanes 2–8: *NotI* digests of genomic DNA derived from: L2 (Canton S, control), L3 (Donor line, control), L4 (*eph^KD^*1), L5 (*eph^KD^*2), L6 (*eph^KD^*3), L7 (*eph^KD^*4) and L8 (*eph^KD^*5). The lower molecular weight band (non-mobilized donor construct) was absent from *eph^KD^* lines 1, 2 & 4 indicating successful homologous recombination. Lanes 9–15: *BglII* digests of genomic DNA derived from: L9 (Canton S, control), L10 (Donor line, control), L11 (*eph^KD^*1), L12 (*eph^KD^*2), L13 (*eph^KD^*3), L14 (*eph^KD^*4) and L15 (*eph^KD^*5). The convergence of distinct donor and endogenous *eph* bands into a single band due to homologous recombination is clearly evident in the *eph^KD^*1, 2 & 4 lines (L11, L12 and L14). C) RT-PCR using primers for the full-length *eph* transcript (L1–9, left side of gel and left diagram; primer locations indicated by arrows). L1 (Canton S, control), L2 (Donor line, control), L3 (*eph^KD^*1/*eph^KD^*1), L4 (*eph^KD^*1/+), L5 (*eph^KD^*2/+), L6 (*eph^KD^*2/*eph^KD^*2), L7 (*eph^KD^*4/+), L8 (*eph^KD^*4/*eph^KD^*4), L9 (*eph^KD^*3/*eph^KD^*3), L10 (molecular weight markers). Full-length transcript was not detected in *eph^KD^* homozygous animals (26 cycles). RT-PCR using primers for the 3′-deleted isoform of Eph is shown in L11–L19, right side of gel and right diagram (primer locations indicated by arrows). Source RNA for L11–L19 was identical to L1–L9. Only the truncated Eph isoform was expressed in *eph^KD^* animals. Abbreviations: LBD (ligand-binding domain), FNIII (fibronectin type III repeats), JXM (juxtamembrane region), TK (tyrosine kinase domain), SAM (sterile alpha motif), PDZ (postsynaptic density 95/Discs-large/zona occludens-1 domain). D,D′) Eph (anti-Eph, red in D, shown alone in D′) is expressed on cortical neuron axons in wild-type third instar larvae, accumulating in a high-midline low-dorsoventral gradient in the medulla neuropil (compare anti-HRP staining, green, to anti-Eph staining, red). E,E′) Lack of Eph immunoreactivity (anti-Eph, red in E, shown alone in E′) corresponded to optic lobe defects (anti-HRP, green) in *eph^KD^* animals, manifest as gaps in the neuropil (arrowheads). F) Schematic of the 5.8 kb *ephrin* locus localized to the 102C2 region of chromosome IV. The *ephrin* gene is comprised of 5 exons (black boxes) and 4 introns (gray lines). The start codon (red arrow) and RS5 P-element insertion site (red shaded box) into 5′UTR of the first exon are also indicated. G,G′) In wild-type animals, Ephrin expression (anti-Ephrin, red in G, shown alone in G′) is punctate along cortical neuron axons and concentrated in the optic lobe neuropil (anti-HRP, green in G). H,H′) In *ephrin^RS5^* mutants, Ephrin expression is considerably reduced in the optic lobe (anti-Ephrin, red in H, shown alone in H′), resulting in neuropil defects (arrowheads; anti-HRP, green in H). White or yellow bars indicate the dorsoventral midline. Scale bar in D is 20 µm for D,D′,E,E′, G,G′, H,H′.

We have previously shown that misexpression of a UAS-*eph^+^* transgene disrupts optic lobe development [21] and reasoned that such transgene-mediated phenotypes might manifest as observable defects in the adult eye. After a survey of *GAL4* driver and transgene combinations, we determined that the moderate rough eye phenotype of *sevenless2-GAL4*, *UAS-ephrin^+^* (hereafter referred to as SE; Figure 2B) animals was best suited for a modifier screen. The SE adult eye defect exhibited little variation of penetrance and was suppressed by co-expression of a dominant-negative *eph* transgene (Figure 2C) or by the *eph^KD^* mutation (data not shown). In the former case, we reason that co-expression with an excess of a kinase-inactive Eph isoform would bind up ectopic Ephrin, suppressing its signaling activity. In the latter case (*eph^KD^*, see Figure 3), the *eph^+^* locus was targeted by homologous recombination to yield a kinase-defective mutant allele. In the absence of a functional Eph kinase, ectopic Ephrin expression had no effect on adult eye morphology. These observations indicate that the SE morphological phenotype is due to ectopic activation of the Eph pathway.

As a preliminary test of the SE background in detecting modifier mutations, deficiency lines representing all four *D. melanogaster* chromosomes (Bloomington *Drosophila* Stock Center) were surveyed for genetic interaction with SE. A total of 196 deficiency lines were screened representing approximately 43% of the *D. melanogaster* genome. Approximately two-dozen enhancers and suppressors of the SE rough eye phenotype were identified by this approach, which gives an estimate of 50–55 interacting loci across the entire genome. To identify point mutations that acted as modifiers, ENU mutagenized animals were crossed into the SE background and the F_1_ progeny examined for enhancement or suppression of the SE eye phenotype (see Materials and Methods). Approximately 15,000 F_1_ progeny were screened in this manner; 52 lines were retained to form a collection of candidate mutants. This collection includes 24 enhancers (13 recessive lethals) and 28 suppressors (11 recessive lethals; see Figure 3D). On the basis of chromosomal location and complementation analysis the 52 mutants were found to define 40 loci, which should represent the majority of interacting loci based on deficiency screen estimates.

### Eph and Ephrin mutants in the characterization of modifier mutations

As a step toward clarifying the developmental roles of candidate genes that function in the Eph/Ephrin signaling pathway(s), we undertook mutagenesis of the *eph* and *ephrin* loci to generate loss-of-function alleles that could be used to establish genetic interactions with the modifier mutations. The *eph* and *ephrin* loci are located on chromosome IV, a small chromosome predicted to harbor about 81 genes. For *eph*, the homologous targeting method [30] was used to introduce a deletion of the kinase domain into the coding region, to create *eph^KD^*. For *ephrin*, we utilized local transposition to introduce a transposon into the 5′ end of the gene. The *eph^KD^* mutation is predicted to be a partial loss-of-function allele, which still retains reverse-signaling and kinase-independent activities. Consistent with this notion, truncated transcripts were expressed in *eph^KD^* mutants (Figure 3 C) while *eph^KD^* phenotypes (Figure 3E) were less severe than those elicited using RNAi [21]. The *eph^KD^* mutation also resulted in increased developmental mortality and female sterility, indicative of requirements for Eph function outside of the optic lobe. The *ephrin^RS5^* mutation also appeared to be a partial loss-of-function allele exhibiting a robust reduction, but not absence of, Ephrin expression (Figure 3H).

The strategy used to generate an Eph receptor mutant with a deleted kinase domain (*eph^KD^*) is depicted in Figure 3A. The ‘donor’ construct generated a tandem partial duplication by ‘ends-in’ recombination to produce one untranslatable copy of *eph* and one translatable copy bearing a 3′ deletion encompassing the entire kinase domain. A total of five independent strains were recovered that displayed mobilization of the donor *w^hs^* gene (Figure 3B). Of these five candidates, three mapped to chromosome IV and were demonstrated to have targeted the *eph* locus by Southern blot analysis. To confirm replacement of endogenous *eph* with the *eph^KD^* isoform, RT-PCR analysis of mRNA transcripts from the *eph^KD^* 1, 2 & 4 lines was performed (Figure 3C). Truncated transcripts predicted to encode the kinase-deleted Eph isoform were detected (Figure 3C), whereas full-length *eph* transcripts were not. Eph immunoreactivity was not detected in *eph^KD^* animals (compare Figures 3D′ and 3E′) by an anti-Eph antibody generated against a C-terminal peptide [21], which would be deleted in *eph^KD^*.

**Figure 4:**
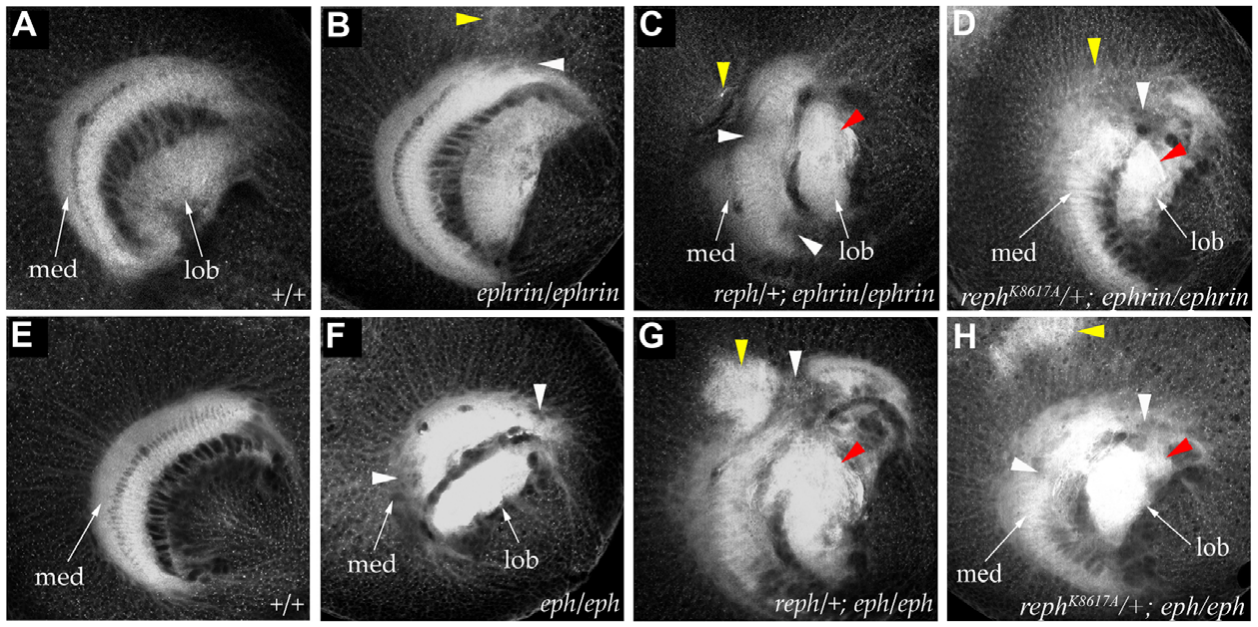
*reph* interacts genetically with the *eph^KD^* and *ephrin^RS5^* mutations. In wild-type flies (A, E) the medulla neuropil (med) is distinguished by its regular crescent shape (anti-HRP staining, grayscale, all panels A–H). Mutant phenotypes could be broadly categorized as defects in the medulla neuropil (white arrowheads), HRP+ cortical inclusions (yellow arrowheads) or disruption of lobula (lob) architecture (red arrowheads). In homozygous *ephrin^RS5^* mutants (B), subtle abnormalities were observed in the medulla neuropil and cortex. The severity of *ephrin^RS5^* medulla defects was enhanced by a single copy of *reph^1^* (C) or *reph^K8617A^* (D). In homozygous *eph^KD^* mutants (F) gaps in the medulla neuropil were often present. A single copy of the *reph^1^* allele (G) or *reph^K8617A^* (H) exacerbated the *eph^KD^* mutant phenotype.

Signaling via the Eph receptor (or ‘forward signaling’) should be abolished in the *eph^KD^* mutant, but ‘reverse signaling’ [40] via the transmembrane Ephrin, whose conserved cytoplasmic tyrosine phosphorylation site is consistent with intracellular signaling functions [36], would still be possible. Homozygous animals displayed reduced viability, with an approximately 50% developmental mortality. Survivors displayed normal external adult morphology. Homozygous *eph^KD^* females exhibited a 60%–90% rate of sterility. Olfactory-based learning in *eph^KD^* animals was also significantly impaired (A. Szybowski and S. Kunes, unpublished observations). Eph signaling is required for normal development of the mushroom body [22], where olfactory learning occurs. Although adult eye structure and gross retinal development were normal in *eph^KD^* animals (data not shown), at the third larval instar stage, when axonal topography is elaborated in the developing adult visual system (Figure 1), *eph^KD^* animals displayed variable defects in the centripetal projections of medulla cortical axons toward the developing neuropil (Figure 3E). These defects appeared similar in nature, albeit less severe, to those that resulted from reduction of Eph expression via RNA interference [21]. The *eph^KD^* animals displayed gaps in the crescent-shaped neuropil and ectopic axon bundles in the cell body layer surrounding the neuropil (Figures 3E and 4E).

To generate a mutation at the *ephrin* locus, we employed local transposition of the P transposon RS5 [29] located adjacent to the *ephrin* locus. The *ephrin* locus was found to be extraordinarily refractory to P-element insertion; however, one local insertion, *ephrin^RS5^*, was obtained that disrupted the 5′ UTR of the *ephrin* mRNA (Figure 3F). The homozygous mutant animals were viable, with normal external adult morphology. They exhibited approximately a 50% reduction in *ephrin* transcript level, as determined by quantitative RT-PCR analysis (data not shown). In wild-type animals, Ephrin is found in puncta on the axons of medulla cortical neurons, and concentrated within the neuropils of the medulla and lobula (Figures 1-C5 and 3G′). Ephrin protein was substantially reduced in the optic lobe of *ephrin^RS5^* homozygotes (Figure 3H′). While these animals displayed normal ommatidial development (data not shown), subtle defects in optic lobe axonal topography were observed (Figure 3H, arrowheads). In the medulla, cortical axon misprojection resulted in neuropil gaps and ectopic axons in the cortical cell body layer. Photoreceptor axon projection defects were observed in the lamina, but these misprojections appeared correlated with defects in medulla architecture. The similarity of the *ephrin^RS5^* hypomorphic phenotype with that of *eph^KD^* is consistent with the notion that these proteins act in the same pathway. However, we must note that many unrelated activities contribute to medulla development and can yield superficially similar axon projection phenotypes.

Although the anti-HRP staining used in these studies doesn't reveal specific aspects of the optic lobe defects associated with *eph^KD^* and *ephrin^RS5^* mutations, such as axon subsets or expression of cell adhesion molecules, it provides useful categorization based on general architectural features. To better assess and characterize these medulla phenotypes, *eph^KD^* and *ephrin^RS5^* animals were scored based on the following criteria: the presence of gaps in the medulla neuropil (0, 1 or >1), the degree of lobula neuropil disruption (none, mild, moderate, or severe) and the presence of HRP^+^ cortical inclusions (yes or no). For *eph^KD^* mutants (n = 16) all exhibited at least one gap in the medulla neuropil with the majority (75%, 12/16) showing more than one such gap. Half of these animals had normal lobula architecture with 38% (6/16) exhibiting mild disruption and only 12.5% (2/16) having moderate-level defects. Large HRP^+^ cortical inclusions were rare, with only 12.5% (2/16) of *eph^KD^* mutants exhibiting this feature. Notably, the *ephrin^RS5^* phenotype appeared qualitatively different based on the aforementioned criteria. In the specimens examined (n = 17), only 12% (2/17) exhibited multiple gaps in the medulla neuropil, while 65% (11/17) had no gaps. Disruption of lobula architecture was more common in *ephrin^RS5^* animals—24% (4/17) exhibited moderate defects and 47% (8/17) showed mild defects. As was the case with *eph^KD^* mutants, large HRP^+^ coritical inclusions were rare in *ephrin^RS5^* mutants, with only 6% (1/17) of the specimens manifesting this phenotype. Thus, the *eph^KD^* phenotype was generally characterized by multiple gaps in the medulla neuropil with relatively normal lobula development, while the *ephrin^RS5^* phenotype was somewhat reversed with a higher percentage of normal medulla development and a greater degree of lobula disruption relative to *eph^KD^* mutants. Such qualitative categorization served as a baseline for assessing genetic interactions between modifier mutations and the *eph^KD^* and *ephrin^RS5^* mutants.

### The SE suppressor mutation, *reph*, interacts with *ephrin* and *eph* mutations

Mutations in genes that participate in Eph/Ephrin signaling should disrupt optic lobe axonal topography as observed when *eph* expression patterns or levels are altered ([21] and below). Such mutations might also have additional distinct phenotypes, as a consequence of the gene's pleiotropic or tissue-specific functions outside of the Eph pathway. The lethal SE enhancers and suppressors displayed a range of temporal lethality, spanning early embryogenesis to late pupation. We examined visual system architecture in homozygous viable and late-lethal mutants at the late third instar larval stage, the time of visual system axon outgrowth and topographic patterning. Of eleven modifier mutations representing distinct loci that were examined at this stage eight displayed defects in medulla architecture (data not shown) roughly similar to *eph* and *ephrin* mutants (Figures 3E and 3H).

Since the criterion of optic lobe developmental defects was insufficient in and of itself to identify a given enhancer or suppressor as a component of the Eph/Ephrin signaling pathway, we sought to establish genetic links. To identify modifier mutations that interacted with the Eph/Ephrin pathway in the establishment of axon topography, we screened the mutant collection for suppression or enhancement of the *ephrin^RS5^* and *eph^KD^* mutant phenotypes (Figures 3E, 3H). This strategy identified an SE suppressor, which we have named *reph* (regulator of *eph* expression, see below) as a strong interactor with Eph pathway function.

*reph^1^* and *reph^K8617A^* were strong suppressors of the SE phenotype, a phenotype induced by ectopic Eph pathway activation (Figure 2). In contrast, heterozygosity for either the *reph^1^* or *reph^K8617A^* alleles enhanced the optic lobe axonal targeting phenotypes of the *ephrin^RS5^* or *eph^KD^* homozygotes (Figure 4), while *reph^1^* or *reph^K8617A^* heterozygosity in an otherwise wild type background displayed normal optic lobe development. All of the phenotypic features associated with the *eph^KD^* were exacerbated by the presence of the *reph* alleles. In the *reph^1^/+; eph^KD^*/*eph^KD^* specimens examined (Figure 4G, n = 10), 80% (8/10) had multiple gaps in the medulla neuropil and all exhibited some form of lobula disruption, 50% (5/10) of these being severe (e.g. multiple gaps, misprojections, etc.). Furthermore, large HRP+ cortical inclusions were frequently observed (8/10 of the specimens examined). For the *reph^1^/+; ephrin^RS5^*/*ephrin^RS5^* genotype (Figure 4C, n = 5), 80% (4/5) exhibited multiple gaps in the medulla neuropil, 80% (4/5) had large HRP+ cortical inclusions, and 80% (4/5) mild to moderate disruption of lobula architecture. Similar phenotypes were observed for the *reph^K8617A^* allele, confirming that the genetic interaction could be attributed to the *reph* mutations and not the genetic background of the lines. As was the case for *reph^1^*, *reph^K8617A^/+; eph^KD^*/*eph^KD^* specimens (Figure 4H, n = 7) exhibited multiple gaps in the medulla neuropil (86%, 6/7), increased lobula disruption (57% or 4/7 being severe) and large HRP+ cortical inclusions (86% or 6/7 of the specimens examined). Likewise, for the *reph^K8617A^/+; ephrin^RS5^*/*ephrin^RS5^* genotype (Figure 4D, n = 5), 80% (4/5) exhibited multiple gaps in the medulla neuropil, 60% (3/5) had large HRP+ cortical inclusions, and 80% (4/5) mild to moderate disruption of lobula architecture. Taken together, these data were consistent with *reph* functioning within the Eph/Ephrin signaling pathway.

### Molecular characterization of the *reph* locus

**Figure 5:**
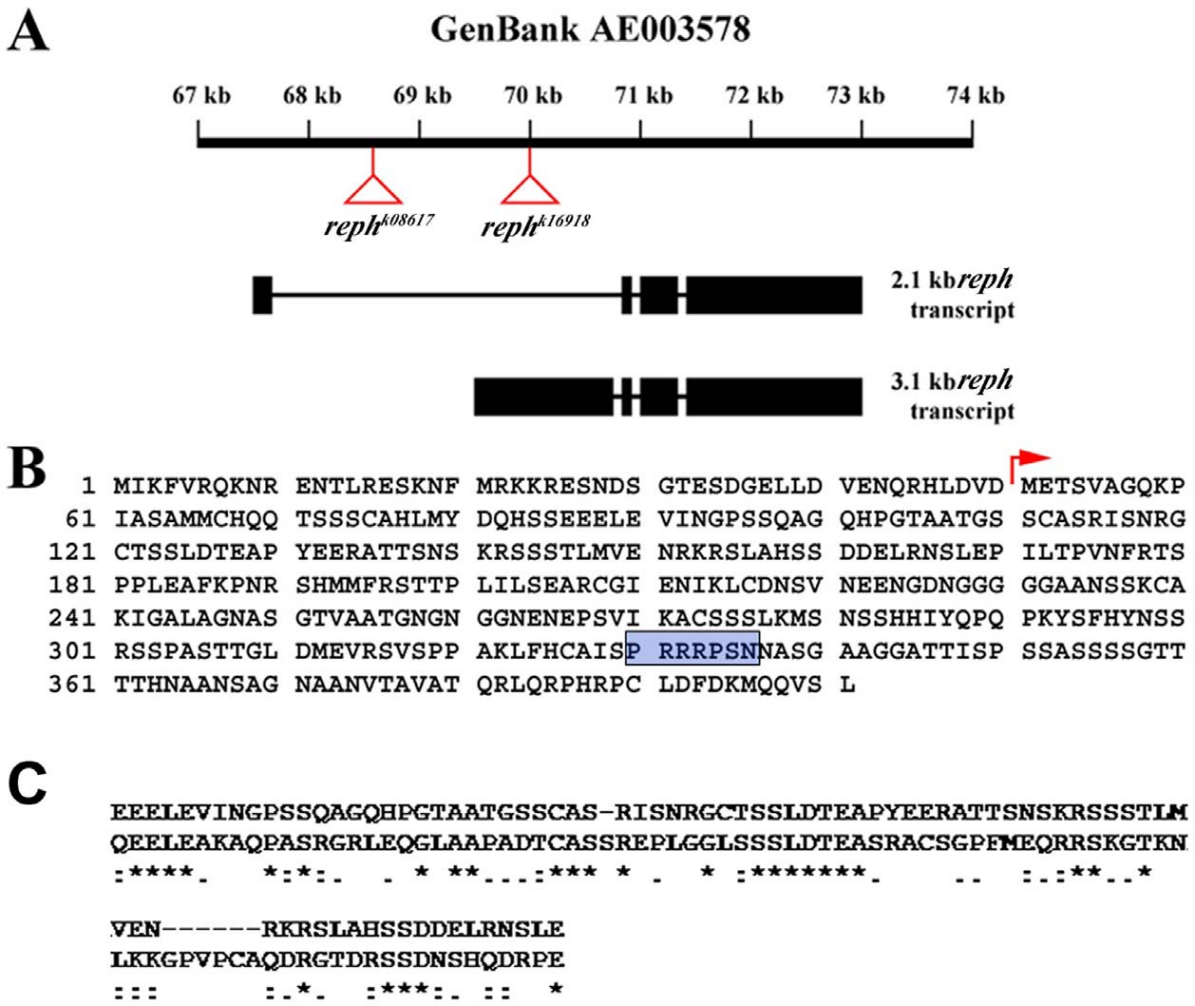
Reph encodes a putative nuclear protein. A) The *reph^1^* allele, *reph^k8617A^*, was mapped by inverse PCR to the CG3920 locus, which harbors another P-element insertion l(2)k16918 (*reph^k16918^*). The *reph* locus spans 5.5 kb and encodes two alternatively spliced transcripts of 2.1 kb and 3.1 kb. Exons are indicated by boxes and introns by connecting lines. The insertion sites for *reph^k8617A^* (first intron of the 2.1 kb transcript) and *reph^k16918^* (5′UTR of exon 1 of the 3.1 kb transcript) are indicated on the GenBank scaffold sequence (AE003578) by red triangles. B) The 3.1 kb transcript is predicted to encode a protein 401 amino acids in length, while the 2.1 kb transcript a protein of 351 amino acids that differ only in the site of translation initiation (red arrow). A conserved nuclear localization signal sequence is indicated by the blue-shaded box. C) Reph shows weak similarities to several transcription factors although with no obvious homologs. An alignment to amino acids 361–451 of the human SPOC-D1 transcription factor (NP 653170) is shown as an example (32% identity, 67% similarity over this stretch).

To gain insight into the role of *reph* in the Eph/Ephrin pathway, we undertook a molecular characterization of the *reph* locus. We used deficiency mapping and complementation with P-element lethal insertion mutations to localize *reph^1^* to *Df(2L)sc19-8*, which spans a small region (24C2-24D2) that contains only 13 predicted genes. Like *reph^1^*, a chromosome bearing *Df(2L)sc19-8* suppressed the SE rough eye phenotype. The *P*-element insertion *P*[*lacW*]*l(2)k16918^k16918^*, one of four lethal insertions uncovered by *Df(2L)sc19-8*, displayed semi-lethality over the *reph^1^* mutant chromosome. DNA sequencing mapped P[*lacW*]*l(2)k16918^k16918^* to the *reph* locus, revealing that the P[*lacW*]*l(2)k16918^k16918^* insertion was indeed a *reph* allele, which was designated *reph^k16918^* (Figure 5A). To generate additional P-element insertions within *reph*, we mobilized a second insertion in the *Df(2L)sc19-8* region, *P*[*lacW*]*bowl^k8617^*,and obtained one *reph^1^* non-complementing insertion, named *reph^k8617A^* (Figure 5A). Rescued DNA sequence flanking the *reph^k8617A^* insertion matched the predicted locus, CG3920 (Flybase). The *reph^k8617A^* allele also suppressed the SE eye phenotype as a *reph^k8617A^*/+ heterozygote (Figure 2E).

The *reph* locus is predicted to encode two mRNAs via alternative splicing (Figure 5A). *reph^k8617A^* is an insertion within the first intron of the 2.1 kb transcript, upstream of the first exon of the *reph* 3.1 kb transcript. The *reph^k16918^* insertion is located 1.3 kb downstream of *reph^k8617A^* within the 5′UTR of the 3.1 kb *reph* transcript. As a final test of the identity of *reph* and CG3920, we examined rescue with a *UAS-reph^+^* transgene (see below). Pan-neural expression of *UAS-reph^+^* with the driver *elav-GAL4* [41] rescued the homozygous lethality of *reph^1^*, consistent with a requirement in the nervous system. Given that *eph* null alleles are viable [22] these data suggest that *reph* has additional functions unrelated to the regulation of *eph*. In addition, SE animals harboring a single copy of *UAS-reph^+^*, under *sevenless2-GAL4* control, displayed a more severe (enhanced) SE eye phenotype (Figure 2F). These data permit the conclusion that *reph* is the CG3920 locus.

The predicted *reph* transcripts encode proteins of 351 and 401 amino acids, which differ in their translation initiation sites (Figure 5B). Reph lacks robust homology to known proteins, although database searches indicate weak similarities between Reph and a variety of transcription factors. An example of such homology to a region of the human SPOC (Spen paralog and ortholog C-terminal) domain-containing 1 protein is shown in Figure 5C. SPOC domain-containing proteins are nuclear effectors of receptor tyrosine kinase signaling in *Drosophila* nervous system development [42]–[44]. Although not itself a member of the SPOC family, Reph harbors a nuclear localization signal sequence, consistent with a putative role in transcription (Figure 5B). Although it lacks any other conserved domain features, the overall structure of Reph may be related to the IMP dehydrogenase/GMP reductase family; the significance of this structural similarity is unclear. Given the limited utility of sequence analysis in revealing *reph*'s developmental role, we turned to more functional studies.

### *reph* expression in the larval third instar visual system

**Figure 6:**
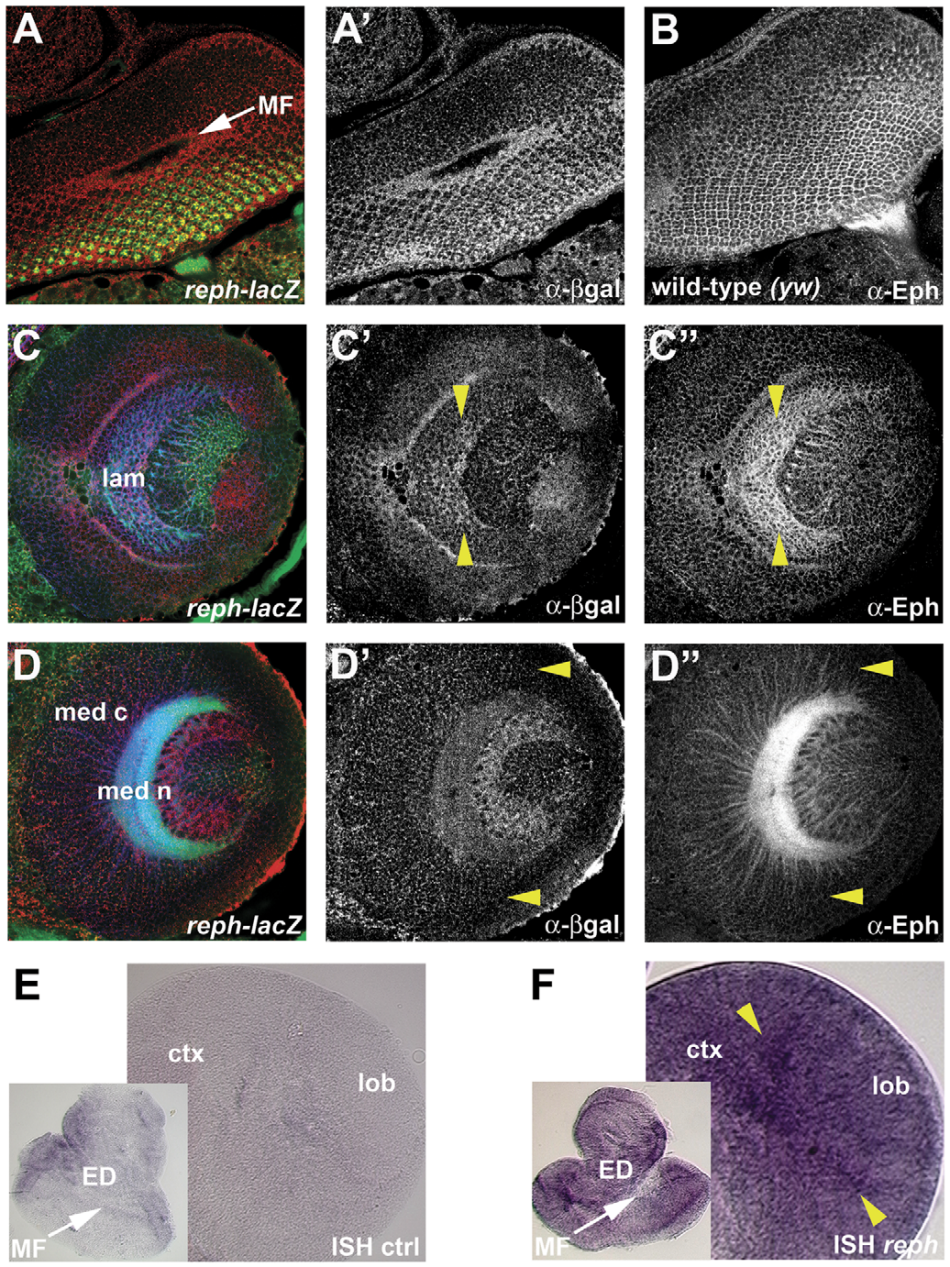
*reph* expression correlates with Eph expression in the developing optic lobe. Expression patterns for *reph* in the developing retina, lamina and medulla were determined using a lac-Z reporter associated with *reph^k8617A^* and by in situ hybridization. Staining for HRP (anti-HRP, green, A,C,D) was used to visualize general cellular architecture. In the developing retina, *reph* (anti-β-gal, red in A, shown alone in A′) was expressed primarily in cells within and posterior to the MF, which correlated well with Eph expression (anti-Eph, B). In the lamina, *reph* was expressed in a high-midline, low-dorsoventral gradient (C′, demarcated by yellow arrowheads), as was the case for Eph (C″). In the medulla, *reph* expression within cortical neurons diminished at the dorsoventral margins (D′, yellow arrowheads) as was the case for Eph expression (D″). In situ hybridization (controls are shown in E) supported the *lacZ* reporter data. In the developing retina *reph* expression was found in cells within and posterior to the MF, while within the lamina region *reph* expression was highest at the midline (F, yellow arrowheads). Abbreviations: cortex (ctx), eye disc (ED), lamina (lam), lobula (lob), medulla cortex (med c), medulla neuropil (med n), morphogenetic furrow (MF).

Developmental expression patterns of *reph* were analyzed via *lacZ* expression from the two *P*[*lacW*] enhancer trap insertions and by in situ hybridization. Reporter expression was found in the eye, lamina, and medulla of the developing visual system at the late third instar stage in patterns overlapping that of Eph (Figure 6). Like Eph, *reph* expression in the eye disc was most pronounced posterior to and within the morphogenetic furrow (Figure 6A′). Within the lamina, *reph* and Eph expression were highest at the posterior midline, diminishing in a graded fashion along the dorsoventral axis (Figures 6C and 6C′). Within the medulla, *reph* expression was similar to Eph, most notably expressed within the Eph-positive cell bodies of the cortex, where cytoplasmic β-galactosidase normally accumulates (Figure 6D′). Within these cortical cells, both *reph* and Eph expression were lowest at the dorso-ventral margins (Figures 6D′ and 6D″, yellow arrowheads). Thus, the graded pattern of *reph* expression in the medulla corresponds to the graded pattern of Eph expression. Additional *reph* expression was found in the lobula where Eph-expressing cells border the posterior face of the medulla (Figures 6D′ and 6D″). Reph *lacZ* expression was additionally examined in the embryonic CNS, where it over-lapped the expression of Eph (data not shown). In situ hybridizations using *reph*-specific probes were also performed on larval third instar brains (Figures 6E and 6F). Although the resolution was low, in situ hybridization showed similar expression patterns as for the *lacZ* reporter lines. Within the eye disc *reph* expression was found in cells posterior to and within the morphogenetic furrow (Figure 6F). Although *lacZ* expression appeared more uniform posterior to the morphogenetic furrow compared with RNA expression, this may be a consequence of β-galactosidase perdurability. In the optic lobe, at regions proximal to the lamina, *reph* expression was highest at the midline (Figure 6F) as was the case for *reph lacZ* expression (Figure 6C′). Taken together, these observations place Reph in the appropriate spatiotemporal context to function in the Eph signaling pathway.

### *reph* is necessary and sufficient for Eph expression in the developing CNS

Animals homozygous for any of the three *reph* alleles failed to survive embryogenesis. These embryos were examined for the expression of Eph in the ladder-like neuropil of the thoracic and abdominal segments. Eph expression was absent, and frequent defects in the ladder-like organization of commissural and longitudinal connectives were observed (data not shown). However, such defects were not observed in *eph^KD^* mutants or in *eph* null mutants [22], which were usually viable. These observations suggest roles for *reph* both in the regulation of Eph expression and in additional activities required for embryonic CNS development and viability.

**Figure 7:**
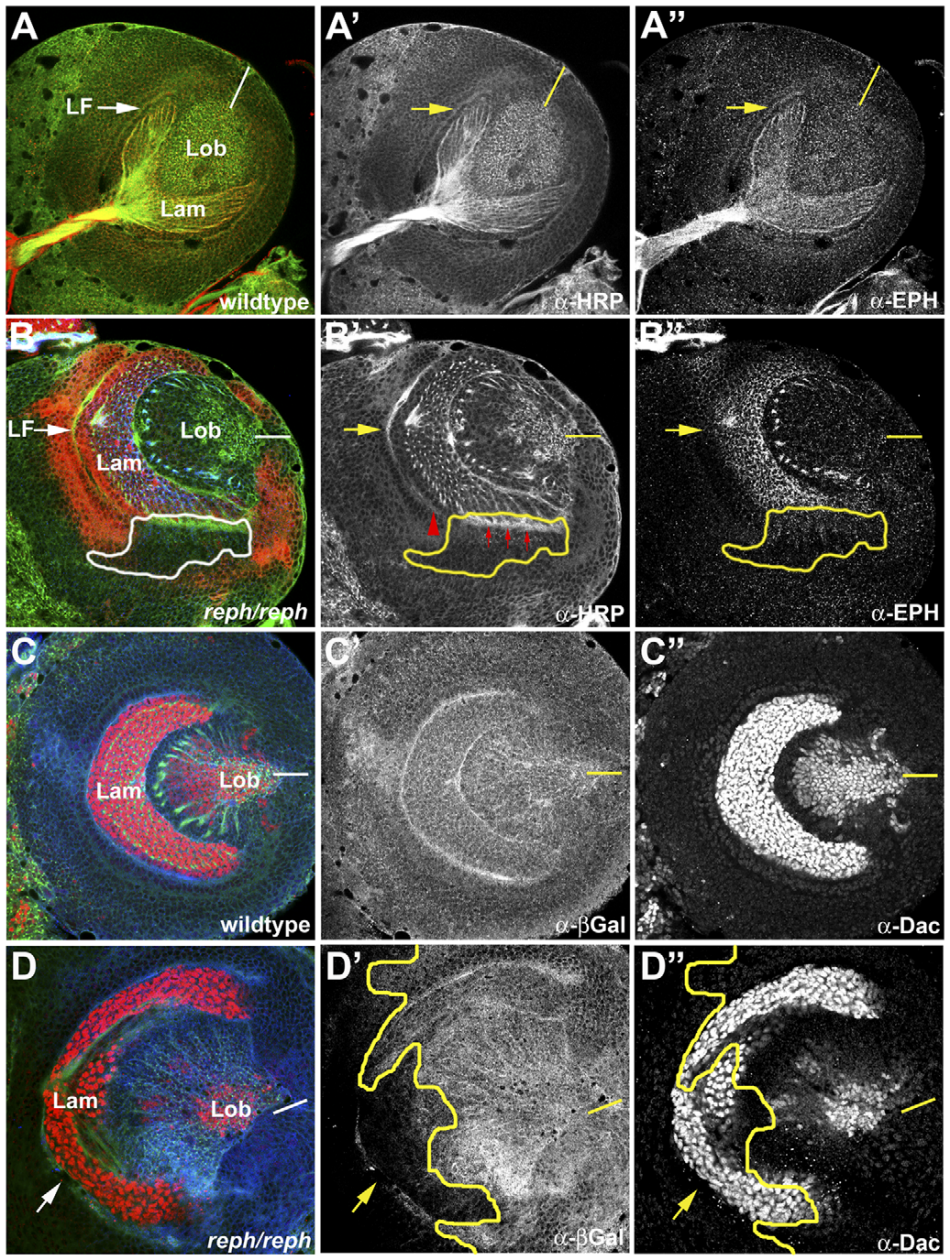
*reph* loss-of-function suggests cell autonomous regulation of Eph expression. A requirement for *reph* in optic lobe development was assessed by generating homozygous somatic *reph^k8617A^* clones by the FLP, FRT method. Mutant clones were marked by loss of expression of an *arm-lacZ* reporter (anti-βgal, red in B, blue in C and D). Overall optic lobe architecture was revealed by anti-HRP staining (green color in all panels, shown alone in A′ and B′). A,A′,A″) A wild type specimen illustrating the normal distribution of Eph expression in the lamina. B,B′,B″) A specimen harboring a homozygous *reph^k8617A^* clone (white or yellow outlines) along the ventral margin of the lamina displayed reduced Eph expression (anti-Eph, blue in B, shown alone in B″) and incomplete LF formation (red arrowhead in B′). To assess potential pleiotrophic effects of reph^k8617A^ on lamina development, reph^k8617A^ clones were stained for dachshund, a marker of lamina neurogenesis (anti-Dac, red color in C and D, shown alone in C″ and D″). C,C′,C″) A wild type specimen highlighting Dac expression in the lamina. D,D′,D″) In the specimen shown, a large reph^k8617A^ clone encompassed most of the ventral lamina (yellow outline) indicated by loss of lac-Z staining (blue). Dac expression was normal within the clone. Dorsal is up and ventral is down in all panels. Abbreviations: lamina (lam), lamina furrow (LF), lobula (lob). Yellow bars in A″–D″ indicate the position of the midline.

In the lamina, Eph is expressed in a gradient, highest in the posterior and lowest at the anterior and dorso-ventral margins (Figure 7A″). Loss of *reph* activity in somatic *reph^k8617A^* clones (Figure 7B, n = 35) reduced Eph expression in the mutant cells (Figure 7B″), but not in their wild type neighbors. Photoreceptor axon projections near such clones were abnormal (red arrows, Figure 7B′) and the lamina furrow failed to extend ventrally through the clone (read arrowhead, Figure 7B′). We considered whether the loss of Eph in the lamina (Figure 7B″) might reflect failure of lamina precursor cells to differentiate [45] by staining *reph^k8617A^* somatic clones for the early differentiation marker Dachshund (Figure 7C″ and [46]). In *reph^k8617A^* somatic clones (n = 7) Dacshund was expressed normally (Figure 7D″), although lamina furrow and photoreceptor projection defects were observed. These data indicated that *reph* acts downstream of lamina induction.

**Figure 8:**
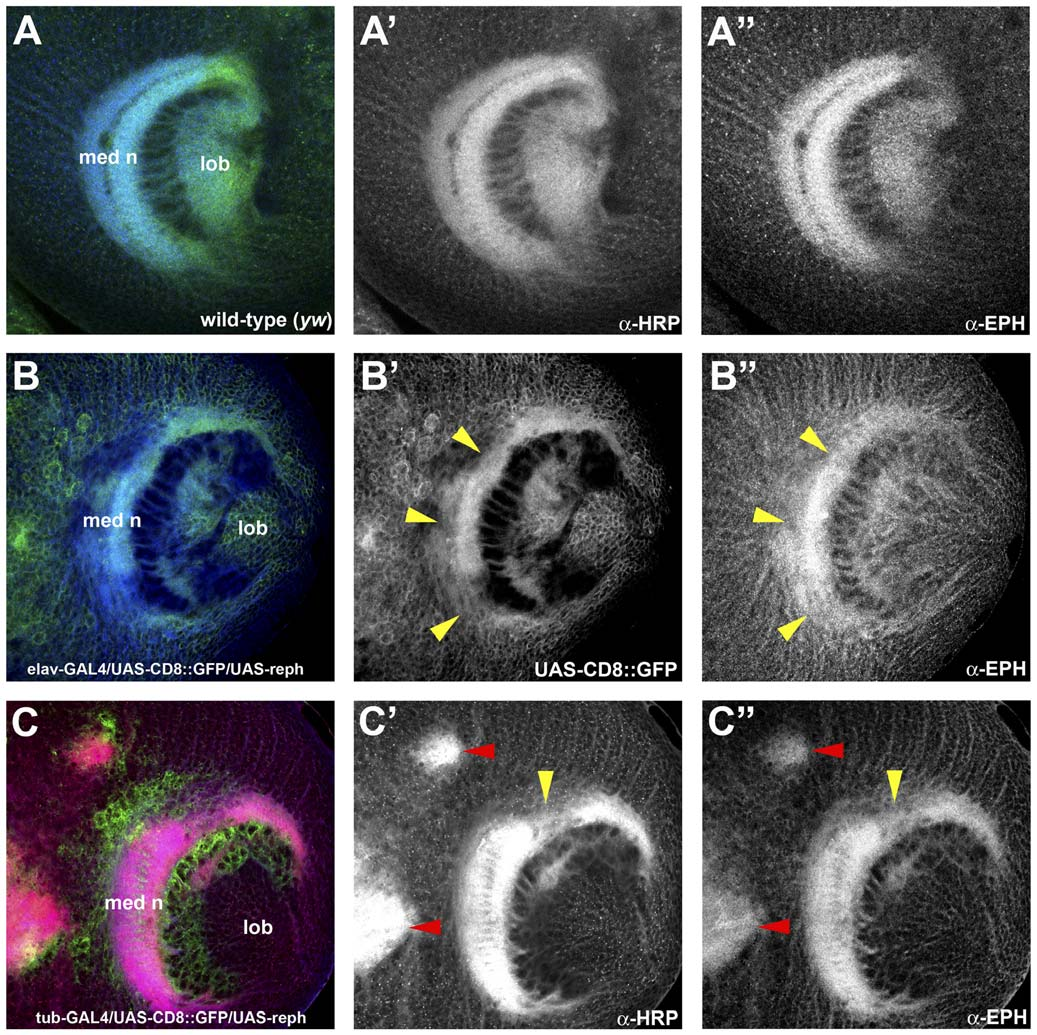
Ectopic misexpression of *reph* up-regulates Eph expression in the developing visual system. To determine whether *reph^+^* was sufficient for Eph expression, the UAS, GAL4 system was used to drive expression of a *UAS-reph^+^* transgene in cell-specific patterns or within somatic clones. GAL4-expressing cells and clones were positively marked by membrane-bound GFP expressed from a *UAS-CD8::GFP* transgene (green color in B and C; shown alone in B′. The axonal architecture was visualized by staining with anti-HRP (green in A, red in C). A,A′,A″) A wild type specimen showing the medulla and its characteristic high midline-low dorsoventral gradient of Eph (anti-Eph, blue in A, shown alone in A″). B,B′,B″) Expression of *UAS-reph^+^* in cortical neurons using an *elav-GAL4* driver flattens the Eph gradient, notably at the dorsoventral margins (anti-Eph, blue in B, shown alone in B″). Defects in medulla development seen as gaps in the neuropil (yellow arrowheads in B′,B″) result from this up-regulation of Eph expression. C,C′,C″) Several *UAS-reph^+^* cortical clones (red arrowheads in C′,C″) generated using a flip-out *tubGAL4* driver can be seen in this specimen. Within these clones, Eph expression (anti-Eph, shown alone in C″) was up-regulated. The enhanced Eph expression was associated with defects manifest as large HRP^+^ cortical inclusions. Disruption of the normal Eph expression pattern also affected medulla neuropil development (yellow arrowhead in C′, C″). Abbreviations: lobula (lob), medulla neuropil (med n).

Conversely, we examined whether *reph^+^* activity was sufficient for Eph expression by using the *UAS*, *GAL4* system [33] to drive ectopic expression of a *reph^+^* transgene encoding the 401 amino acid isoform of Reph (see Materials and Methods for details). Whether the smaller (351 amino acid) Reph isoform differs in activity and/or function remains to be examined. Eph is expressed in subsets of developing neurons; within the medulla, the graded pattern of Eph expression (high midline, low dorsoventral) is particularly notable (Figure 8 A″). We first examined how driving uniform *reph^+^* expression, using the pan-neural *elav-GAL4* driver [47], affected both Eph expression patterns and medulla architecture (Figure 8B). As can be seen in Figure 8B″, *elav-GAL4* driven expression of the *reph^+^* transgene effectively leveled Eph expression throughout the medulla, eliminating the characteristic gradient—this was most noticeable at the dorso-ventral margins. As a consequence, medulla architecture was abnormal, manifest as gaps in the medulla neuropil (arrowheads, Figure 8B′) similar to those observed when Eph/Ephrin signaling was disrupted in the *eph^KD^* and *ephrin^RS5^* mutants. To refine the ectopic expression approach, a “FLP-out” activated *GAL4* driver, P[*tub_α1_*>*y+*,*CD8*>*GAL4* ] [48], was used to express *UAS-reph^+^* in somatic cell clones (Figure 8C). Clones were positively marked by the co-expression of *UAS-CD8*::*GFP* [49]. In specimens harboring clones within the medulla, GFP-positive clones displayed enhanced Eph expression and were associated with areas of large cortical inclusions (red arrowheads, Figures 8C′ and 8C″). Gaps within the medulla neuropil, an indication of axon projection defects, were also observed (yellow arrowhead, Figures 8C′and 8C″). Taken together, these data indicate that *reph* functions upstream of Eph as a regulator of its expression.

## Discussion

In vertebrates, the diverse array of Eph and Ephrin family members contribute to many aspects of nervous system development, notably axon guidance [1], [3], [50]. Surprisingly, although expressed throughout the embryonic CNS [36], [51] and larval visual system [21], the contribution of the single *D. melanogaster* Eph receptor/Ephrin ligand pair in terms of axon guidance and overall nervous system development remains unclear. While RNAi knock-down experiments have suggested a role for this pathway in axon guidance [21], [36], analyses of an Eph null allele have demonstrated little to no role in axon guidance within either the developing embryonic or larval nervous system but rather a specific requirement for mushroom body development [22]. Since the *ephrin^RS5^* and *eph^KD^* mutants described in this paper exhibit axon guidance defects reminiscent of RNAi-induced phenotypes, how Eph/Ephrin signaling is translated into functional outcomes remains unclear but would seem to depend on the nature of the allele (e.g. null, forward-signaling inhibited, etc.). Thus, the 40 interacting loci that potentially encode Eph pathway components recovered in the genetic screen described in this paper are likely to help shed light in many areas of Eph/Ephrin signaling. One of these, *reph*, encodes a putative nuclear factor that evidently regulates Eph expression.

### Identification of Eph/Ephrin-interacting molecules via genetic screening

Using the now conventional modifier screen based on an externally visible eye defect [23]–[25] we identified a group of candidate genes encoding components of the Eph/Ephrin signaling pathway. The SE phenotype used in this screen (Figure 2B) presumably resulted from hyper-activation of Eph signaling in the retina during development because it was suppressed by co-expression of a C-terminal truncated, dominant-negative Eph isoform or by the *eph^KD^* mutation. Of the 52 modifiers recovered representing 40 loci, 24 carried lethal mutations. Since *eph* null alleles are viable [22] it is likely that the genes associated with these lethal mutations have additional developmental functions distinct from their roles within the Eph/Ephrin signaling pathway. Although many of the mutants (8/11 loci examined, data not shown) that survived to the late third instar larval stage displayed optic lobe defects consistent with a role in the Eph pathway, such phenotypes alone could not be used to establish that the affected genes were components of the Eph/Ephrin signaling pathway, given the diverse cellular processes and molecular pathways involved in optic lobe development. Therefore, evidence for genetic interaction between modifier mutations and Eph/Ephrin mutants was investigated.

### Allele-specific effects of Eph/Ephrin mutations on *Drosophila* nervous system development

*D. melanogaster* encodes a single Eph receptor/Ephrin ligand pair, expressed in the embryonic CNS, the developing visual system (Figure 1) and the adult brain. Despite the simplicity of this single pairing in *D. melanogaster*, the role of Eph/Ephrin signaling with respect to nervous system development is surprisingly complex. Although functional as a canonical receptor/ligand pair when ectopically activated in both the embryonic CNS [22], [36] and developing visual system [21], Eph and Ephrin mutations, exhibit both tissue selective and allele-specific effects, instead of revealing a generalized role. Eph-null mutations [22] and a mutation that deletes the Eph kinase domain (*eph^KD^*; Figure 3) are without discernible effect on the embryonic CNS. However, both the *eph^KD^* and *ephrin^RS5^* alleles exhibited defects in visual system development (Figure 3). Surprisingly, prior examination of an Eph null mutation did not reveal photoreceptor axon guidance defects in the developing visual system [22]. However, a requirement for Eph-signaling in the olfactory system is revealed by both Eph-null and *eph^KD^* mutants; axon guidance of mushroom body neurons is affected in the Eph-null background [22] while *eph^KD^* flies exhibit defects in olfactory-based learning (A. Szybowski and S. Kunes, unpublished observations) that might be accounted for by developmental abnormalities.

The distinctions between these observations may result from underlying differences in the individual mutant genotypes and together suggest that the degree of Eph-specific and Ephrin-specific signaling plays a critical role in axon guidance outcomes. Neither Eph nor Ephrin signaling would be activated in Eph-null mutations, but the *eph^KD^* allele encodes a truncated polypeptide capable of activating Ephrin signaling. In vertebrates, kinase-truncated Eph receptors are active as ligands for signaling through transmembrane B-type Ephrins [52], [53], a class that may include the *D. melanogaster* Ephrin [36]. Thus, activation of Ephrin signaling while simultaneously inhibiting Eph signaling, as is the case for the *eph^KD^* allele, may produce developmental outcomes distinct from failure to activate either arm of the receptor/ligand pair as would be the case for Eph-null mutations. Indeed, the fact that Eph and Ephrin can signal independently even within the same cell [38] supports the notion that integration of these distinct signaling arms may be critical determinants of Eph/Ephrin signaling outcomes.

Defects in visual system development for *eph^KD^* mutants were qualitatively similar to, albeit less severe than, the loss-of-function phenotype induced by RNAi knockdown of Eph [21], the most consistent of which were defects in cortical axon guidance (Figure 3). There were additional defects noted with *eph^KD^*, such as reduced viability and sterility; these phenotypes likely were not detected in the prior analysis because they are also outcomes of microinjection, which was used to introduce double-stranded RNA. A synthesis of these observations indicates that cortical axon guidance in the developing optic lobe requires the kinase-dependent ‘forward’ signaling activity of Eph in the context of simultaneous Ephrin activation. The abundance of cortical axon guidance defects associated with putative Eph signaling mutants (data not shown) supports this conclusion. Roles for Eph signaling beyond axon guidance during nervous system development await further elucidation. The slightly weaker, similar phenotypes observed in the mutant *ephrin^RS5^* (Figure 3) are consistent with the notion that Ephrin plays the role of ligand in the optic lobe, inducing Eph kinase activity. While detailed characterization of the *eph^KD^* and *ephrin^RS5^* mutants remains, their moderate phenotypes were well-suited to the purpose of screening for genetic interactions amongst the collection of candidate Eph pathway genes.

### Reph, a novel regulator of Eph expression

A suppressor mutant, designated here as *reph* (regulator of Eph expression; Figure 5), exhibited genetic interactions with *eph^KD^* and *ephrin^RS5^* mutants (Figure 4), consistent with function in Eph/Ephrin signaling. *reph* mapped to the region 24C2-24D2 by complementation analyses using chromosomal deficiencies and lethal P-element insertions localized *reph* to the CG3920 locus, which encodes two transcript isoforms (Figure 5). Ectopic expression of CG3920, in the form of a *UAS-reph^+^* transgene, rescued *reph* mutant phenotypes, verifying the identity of *reph* as CG3920. The single most prominent feature of Reph is the presence of a nuclear localization sequence, P_330_RRRPSN_336_, suggesting a putative nuclear protein. Alignment searches indicate that Reph has similarities to a variety of transcription factors, such as the human SPOC-D1 protein (Figure 5), although no obvious homologues. Consistent with a role in transcription, Reph does appear to act as a positive regulator of Eph expression in the optic lobe.

All *reph* alleles, and heteroallelic combinations, were found to be recessive or semi-lethal, with the majority of animals dying during embryogenesis. The viability of both Eph-null mutations [22] and *eph^KD^* mutants (Figure 3) suggests that *reph* has additional functions during embryonic development aside from the regulation of Eph expression. Elucidating the extent of *reph*'s developmental role, particularly the control of Reph expression itself, is of obvious future interest.

Throughout the late third instar optic lobe, *reph* expression was correlated with Eph expression (Figure 6), placing Reph in the correct spatio-temporal context to regulate Eph expression. Both the necessity and sufficiency of Reph in the regulation of Eph were demonstrated via loss-of-function/gain-of-function experiments. When *reph* function was eliminated in somatic clones, Eph expression was reduced in a cell-autonomous fashion, whereas strong ectopic *reph^+^* expression produced a cell-autonomous increase in Eph expression. Both perturbations resulted in developmental defects corresponding to the mutant clones (Figures 7 and 8). It is unclear to what extent these observed phenotypes generated by disruption of *reph* expression are attributable to changes in Eph and how much might be due to additional *reph* functions. The embryonic lethality of *reph* alleles, but not *eph* nulls, suggests such additional *reph* functions. Furthermore, *reph* expression within the medulla appears more extensive than that of *eph* (Figure 6), which could indicate functions outside of the Eph/Ephrin signaling pathway. However, the effects of *reph* loss-of-function on optic lobe development were consistent with established *eph* mutant phenotypes. Additionally, differentiation of cells within *reph* somatic clones was normal, even though Eph expression was decreased (Figure 7). These data indicate that, at least in the optic lobe, *reph* is a novel regulator *eph* expression.

### Regulation of Eph/Ephrin expression

Eph/Ephrin signaling has been most thoroughly characterized in terms of growth cone dynamics. Less is known, by comparison, about the regulation of Eph receptor and Ephrin ligand expression patterns. Homeobox transcription factors regulate the expression of EphA receptors during rhombomere boundary formation in the vertebrate hindbrain [54]–[56] and developing retina [57]. Within the optic tectum, *engrailed* family members regulate the expression of Ephrins [58]–[60]. In the retina, the T-box transcription factor Tbx5 and Vax homeodomain proteins reciprocally control the expression of B-type Eph receptors and Ephrins along the dorsal-ventral axis [61], [62]. Homeobox genes also regulate EphB receptors during vascular development [63]. The identification of Reph expands the repertoire of transcriptional regulators of Eph expression. Further study of Reph function is anticipated to shed light on the regulation of Eph expression patterns, which is vital given the numerous biological processes mediated by Eph/Ephrin signaling in developing and adult tissues. We anticipate that the identification and characterization of additional genes recovered in the modifier screen will elucidate temporal-spatial specific determinants governing Eph/Ephrin signaling outcomes and facilitate a deeper understanding of the evolutionary conserved mechanisms through which this receptor/ligand pair operates.
